# The Phase-Dependent Regulation of Lux-Type Genes on the Spoilage Characteristics of *Hafnia alvei*

**DOI:** 10.3390/foods13050688

**Published:** 2024-02-24

**Authors:** Jingran Bi, Qiaoli Yao, Gongliang Zhang, Hongman Hou

**Affiliations:** 1 School of Food Science and Technology, Dalian Polytechnic University, No. 1, Qinggongyuan, Ganjingzi District, Dalian 116034, China; bijingran1225@foxmail.com (J.B.); yaoql9393@foxmail.com (Q.Y.); zgl_mp@163.com (G.Z.); 2 Liaoning Key Lab for Aquatic Processing Quality and Safety, No. 1, Qinggongyuan, Ganjingzi District, Dalian 116034, China

**Keywords:** quorum sensing, spoilage, biogenic amines, self-organizing map analysis

## Abstract

*Hafnia alvei*, a specific spoilage microorganism, has a strong capacity to destroy food protein and lead to spoilage. The aim of this study was to evaluate the phase-dependent regulation of lux-type genes on the spoilage characteristics of *H. alvei* H4. The auto-inducer synthase gene *luxI* and a regulatory gene *luxR* of the quorum sensing systems in *H. alvei* H4 were knocked out to construct the mutant phenotypes. On this basis, the research found that the *luxI* and *luxR* genes had a strong positive influence on not only flagella-dependent swimming ability and biofilm formation but also the production of putrescine and cadaverine. The *luxR* gene could downregulate putrescine production. The maximum accumulation of putrescine in wild type, Δ*luxI*, Δ*luxR* and Δ*luxIR* were detected at 24 h, reaching up to 695.23 mg/L, 683.02 mg/L, 776.30 mg/L and 724.12 mg/L, respectively. However, the *luxI* and *luxR* genes have a potential positive impact on the production of cadaverine. The maximum concentration of cadaverine produced by wild type, Δ*luxI*, Δ*luxR* and Δ*luxIR* were 252.7 mg/L, 194.5 mg/L, 175.1 mg/L and 154.2 mg/L at 72 h. Moreover, the self-organizing map analysis revealed the phase-dependent effects of two genes on spoilage properties. The *luxI* gene played a major role in the lag phase, while the *luxR* gene mainly acted in the exponential and stationary phases. Therefore, the paper provides valuable insights into the spoilage mechanisms of *H. alvei* H4.

## 1. Introduction

Food spoilage is mainly a consequence of the degrading enzymatic activity triggered by some spoilage microorganisms. For the spoilage microorganism, its excellent flagella-dependent swimming ability could help access an appropriate niche inside food, and strong biofilm formation could enhance the bacterium’s ability for colonizing a food surface, leading to resistance to antibacterial agents and food processing conditions [1]. Moreover, the spoilage microorganism has a potential ability to produce massive metabolic end products, such as saccharolytic, proteolytic, pectinolytic and lipolytic enzymes, leading to food spoilage [2]. Generally, decarboxylase-positive microorganisms are mainly involved in the decarboxylation of amines to produce biogenic amines (BAs), giving food an undesirable taste and “putrid odor” and affecting public health [3,4].

*Hafnia alvei*, a specific spoilage microorganism, has a strong capacity to destroy food protein, leading to spoilage. It is a Gram-negative bacterium with *lux*-type quorum sensing (QS) systems. Most genetic strains can grow over a wide range of temperatures, even at a minimum temperature of 0.2 to 3.7 °C [5]. With a psychrotrophic attribute, *H. alvei* has the opportunity to be dominantly found in spoiled food, including vacuum and air-conditioned packaged food and cryopreservation food, such as dairy [6], fish [7,8], meat [9] and other protein-rich food. Although many studies have shown that *H. alvei* is considered to be a typical contributor to spoilage with the production of BAs [10], the self-regulating mechanisms of spoilage characteristics in *H. alvei* are still in their infancy.

QS is a common regulatory mechanism of biological functions in the microorganism kingdom [11,12]. QS plays a key role in coordinating group behavior and addressing changes in external and internal environments [13,14]. That is to say, QS is a comprehensive regulator of various biological aspects of microbe metabolism processes [15]. The *lux*-type system is a typical kind of QS system with an auto-inducer (AI) signaling molecule for mediating intracellular signal recognition and is commonly found in *H. alvei* [16]. Two essential genes mainly accomplish the pathways of QS regulation: *luxI*, an AI synthase gene; and *luxR*, a regulatory gene [17]. The *luxI* gene can regulate the synthesis of AI and *luxR* can regulate reception with AI, which has an important influence on the expression of the target functional genes, including virulence modulation, exoenzyme enzyme synthesis and biofilm formation [18]. Therefore, we supposed that the *luxI* and *luxR* genes also have the potential ability to regulate spoilage behaviors.

The aim of this study was to determine the phase-dependent effect of *luxI* and *luxR* genes on spoilage characteristics in *H. alvei* H4. Through *luxI* and *luxR* gene knockouts, mutant phenotypes were constructed. Furthermore, growth ability, swimming motility, biofilm formation and BA formation capacity between wild and mutant strains were comparatively studied by self-organizing map (SOM) analysis.

## 2. Materials and Methods

### 2.1. Bacterial Strains and Reagents

Bacterial strains and plasmids used in this study are listed in Table 1. *H. alvei* H4 was cultured in Luria–Bertani (LB) (10 g tryptone, 5 g yeast extract power, 10 g NaCl, dissolved in 1 L deionized water) agar plates at 37 °C. The molecular biology reagents and precursor amino acids including L-histidine monohydrochloride monohydrate, L-tyrosine disodium salt hydrate, L-ornithine monohydrochloride, L-lysine monohydrochloride, L-tyrosine disodium salt hydrate, L-arginine monohydrochloride, L-phenylalanine hydrochloride and L-tryptophan hydrochloride were obtained from Sangon Biotech (Shanghai) Co., Ltd. (Shanghai, China). Acetonitrile used for high-performance liquid chromatography (HPLC) was chromatographically pure, and other chemical reagents used in this study were of analytical grade; all of them were purchased from Bonuo biochemical reagent Co., Ltd. (Dalian, China). Tryptone, yeast extract power, NaCl, agarose, glucose, Tween-80, MgSO_4_, MnSO_4_, FeSO_4_, ammonium citrate, thiamine, K_2_PO_4_, CaCO_3_, pyridoxal-5-phosphate and bromocresol purple were also purchased from Bonuo biochemical reagent Co., Ltd. (Dalian, China).

### 2.2. Construction of luxI and luxR Mutants and Complemented Strains

For characterizing the QS system in *H. alvei* H4, the *luxI*, *luxR* and *luxIR* gene knockout mutants and their complemented strains were constructed according to a report by Zhu et al. [21]. The *luxI*-F/R and *luxR*-F/R primers were designed as in Table 2 by Primer 3.0. The flanking regions of *luxI* and *luxR* were amplified by PCR with the primer pairs using pfu DNA polymerase. After confirming the sequence, the upstream and downstream regions of *luxI* with 628 bp and 654 bp, as well as *luxR* with 608 bp and 659 bp were digested by EcoRI/BamHI and BamHI/SphI, respectively, cloned into the plasmid pUC19 to create pUC19-Δ*luxI*, pUC19-Δ*luxR* and pUC19-Δ*luxIR*, and then introduced into *E. coli* TOP10 for identification by PCR. Subsequently, the plasmids were linked by a BamHI restriction site and the *luxR*, *luxI* and *luxIR* gene regions were replaced with a chloramphenicol resistance gene cassette (Cm^R^) (1048 bp) previously amplified from the donor plasmid pKD3 using primers Cm-F/R, respectively. The plasmids including pUC19-Δ*luxI*::Cm, pUC19-Δ*luxR*::Cm and pUC19-Δ*luxIR*::Cm were created, then the target fragments, such as *luxI*::Cm, *luxR*::Cm and *luxIR*::Cm, were digested by SalI and inserted into the same sites of the suicide plasmid pCVD442 to acquire the recombinant plasmids pCVD442-Δ*luxI*::Cm, pCVD442-Δ*luxR*::Cm and pCVD442-Δ*luxIR*::Cm. The recombinant plasmids were introduced into *E. coli* β2155 (donor strain) by electroporator. *E. coli* β2155 harboring three different plasmids as pCVD442-Δ*luxI*::Cm, pCVD442-Δ*luxR*::Cm and pCVD442-Δ*luxIR*::Cm were conjugated with wild-type *H. alvei* H4, respectively. Recipient cells were plated on LB supplemented with 50 μg/mL Amp, 0.5 mM DAP and 10 μg/mL antibiotic chloramphenicol to select the successful clone recombinant plasmids that had integrated the vector by a single crossover of allelic exchange. Antibiotic-resistant colonies were selected and confirmed by PCR. The *luxR*, *luxI* and *luxIR* gene knockout mutant phenotypes were named as Δ*luxI*, Δ*luxR* and Δ*luxIR*, respectively.

To build the corresponding complementary plasmids for the Δ*luxI*, Δ*luxR* and Δ*luxIR* mutants, the *luxI* and *luxR* genes were amplified by primers as before, and then the fragments were cloned into plasmid pET28a(+)/FaGH17A with kanamycin resistance to construct pET28a(+)/FaGH17A-Δ*luxI*, pET28a(+)/FaGH17A-Δ*luxR* and pET28a(+)/FaGH17A-Δ*luxIR*. The plasmids were primarily transformed into *E.coli* TOP10 and plasmid DNA was isolated and then transformed into the Δ*luxI*, Δ*luxR* and Δ*luxIR* mutants to produce the complementary strains *comI*, *comR* and *comIR* whose presence were confirmed by PCR analysis and sequencing.

### 2.3. Growth Curve

The growth of wild, mutant and complementation phenotypes was measured by optical density at OD_600_ nm every 6 h via an ultraviolet spectrophotometer. For comparative analysis of growth kinetics, the Gompertz model [22] was applied to fit the OD_600_ data. Per the parameters including maximum specific growth rate (V_max_), lag time (Lag) and maximal OD_600_ at stationary phase (A_max_), the growth abilities of different strains were compared.

### 2.4. Swimming Motility Assay

As in a previous report [23], the swimming motility of wild, mutant and complementation phenotype strains were measured with some modifications. Briefly, 3 μL of the overnight cultured strain was placed in the center of a swimming agar plate including 1% tryptone, 0.5% NaCl and 0.3% agarose. After incubating at 30 °C for 48 h, the migration distances of different phenotypes were recorded by measuring the diameters of the colony zones.

### 2.5. Biofilm Formation Assay

According to the experimental method reported by Liu et al. [24], the biofilm formation abilities of *H. alvei* H4 strains were evaluated. Briefly, 200 μL of wild, mutant and complementation phenotypes were incubated at 30 °C for 48 h in a 96-well plate. Then the culture suspension was removed and the plate was rinsed thrice with PBS (pH 7.4, 0.01 M) and 200 μL methanol and 200μL of 0.1% crystal violet were added for immobilization and as dye. The plate was again rinsed thrice with deionized water and dried at 60 °C. The biofilm was extracted using 200 μL 33% acetic acid followed by a 20 min incubation at room temperature. The absorbance was recorded at 590 nm with an ultraviolet spectrophotometer.

### 2.6. Decarboxylase Detection

Based on Chang’s work [25], the decarboxylase production abilities of H. alvei H4 strains were estimated with some modifications. Briefly, one colony of wild-type, Δ*luxI*, Δ*luxR* and Δ*luxIR* strains were cultivated overnight in 5 mL of LB broth. Then 1 mL of culture was added to 9 mL of the decarboxylase media (LB supplemented with 0.05% glucose, 0.1% Tween-80, 0.02% MgSO_4_, 0.005% MnSO_4_, 0.004% FeSO_4_, 0.2% ammonium citrate, 0.001% thiamine, 0.2% K_2_PO_4_, 0.01% CaCO_3_, 0.005% pyridoxal-5-phosphate, 0.006% bromocresol purple, 2% aga) in a screw-cap test tube containing 0.1% precursor amino acid and cultivated for 24 h. Then the chromogenic reaction of the mixture was observed.

### 2.7. HPLC Analysis of BA Production

The determination of BAs was conducted based on the work of Wang et al. [26]. *H. alvei* H4 strains (wild type, Δ*luxI*, Δ*luxR* and Δ*luxIR*) were cultivated in LB supplemented with 0.005% pyridoxal-5-phosphate and 0.1% precursor amino acid for 24 h. Then 1 mL of culture was mixed with 9 mL 10% trichloroacetic acid in a centrifuge tube. After standing for 2 h at 4 °C, the mixture was homogenized for 10 min (3000× *g*). A 200 μL volume of supernatant was derivatized using 80 μL 2 mol/L NaOH and 800 μL 10 mg/mL dansyl chloride dissolved in acetone. After water-bath heating at 45 °C for 40 min, 50 μL ammonium hydroxide and 550 μL acetonitrile were added into the dansyl derivatives, homogenized for 5 min (3000× *g*) and filtrated through a 0.22 μm filter. Finally, 10 μL aliquots were injected for HPLC analysis.

The concentrations of BAs were determined by an HPLC system (ZORBAX, Agilent, Tokyo, Japan). An SB-C_18_ reversed-phase column (5 μm, 4.6 mm × 125 mm; Agilent, Tokyo, Japan) was used for chromatographic separation. The gradient elution program was operated with acetonitrile/water as the mobile phase.

### 2.8. Statistical Analysis

Each sample was subjected to three replicate trials, and all experiments were repeated three times. Results were presented as mean standard deviation (SD) and analyzed by t-test using SPSS 16.0 software, assuming statistical significance at *p* < 0.05. The Self-Organizing Map (SOM) was established by HMM toolbox (MATLAB7.8, The Math Works, R2009) to classify data patterns of the putrescine and cadaverine productivity of wild type, Δ*luxI*, Δ*luxR* and Δ*luxIR*. All of the graphs were made by origin version 8.0.

## 3. Results

### 3.1. Mutant and Complementation Strains Construction

**Figure 1:**
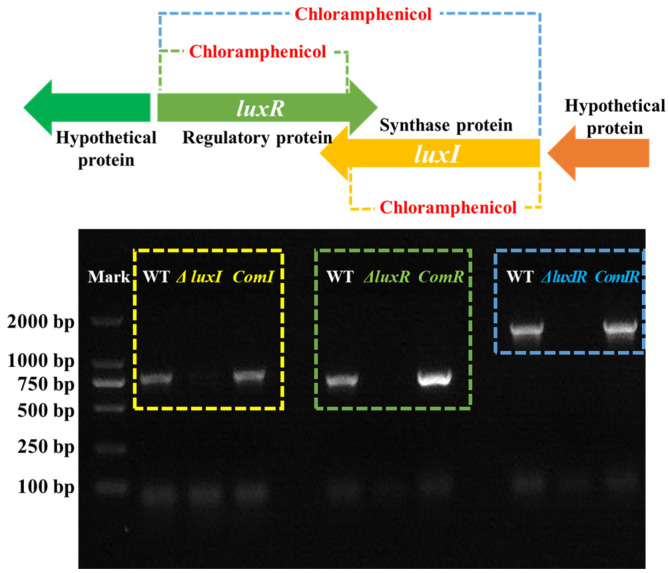
PCR analysis of the mutant and complementation phenotypes.

*H. alvei* coordinates communal behavior as a function of population density by *lux*-type QS systems. This mechanism typically involves N-acyl homoserine lactones (AHLs), a kind of AI signaling molecule, described as a “language” of cell-to-cell communication, which is used by *H. alvei* to understand changes in its environment and consequently to apply specific strategies that allow adaptation to environmental stress in space and time [27]. The synthesis of AHLs is regulated by *luxI*, an AHL synthases gene. The sense of AHLs is regulated by *luxR*, a response transcriptional regulator gene. This could potentially lead to multiple target spoilage behaviors being regulated. For characterizing the QS system in *H. alvei* H4, the *luxI*, *luxR* and *luxIR* gene knockout mutants and their complemented strains were constructed. Through PCR analysis (Figure 1), the bands corresponding to the *luxI*, *luxR* and *luxIR* genes in the Δ*luxI*, Δ*luxR* and Δ*luxIR* mutant phenotypes, respectively, were not detected due to the target fragment being replaced with chloramphenicol. Furthermore, the corresponding lost bands recurred in the complementation strains, *comI*, *comR* and *comIR*, which is attributed to the recovery of the *luxI*, *luxR* and *luxIR* genes.

### 3.2. Growth Ability, Swimming Motility and Biofilm Formation

**Figure 2:**
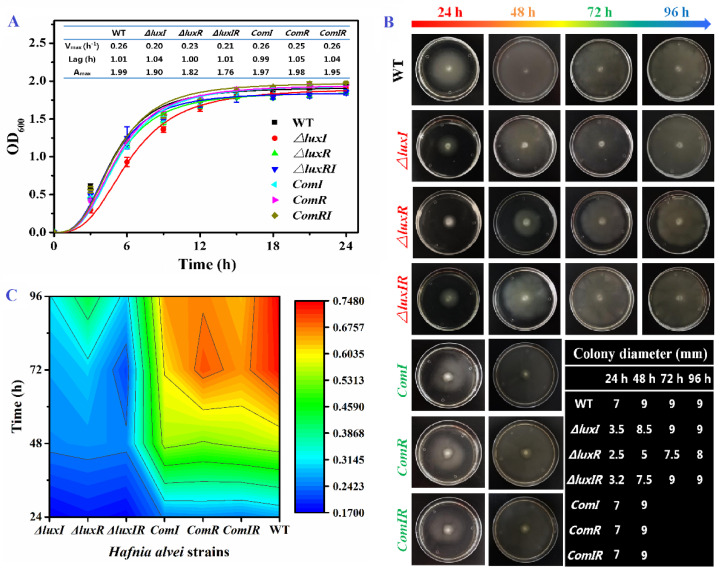
Characterizations of wild, mutant and complementation phenotypes. (**A**) Growth curve fitted with the Gompertz model. (**B**) Swimming motility. (**C**) The contour of biofilm formation.

The *luxI*, *luxR* and *luxIR* gene knockout mutant phenotypes of *H. alvei* H4 were successfully constructed and their growth ability, swimming motility and biofilm formation were investigated (Figure 2). As shown in Figure 2A, the growth curves of *H. alvei* H4 including wild, mutant and complementation phenotypes were obtained and fitted by Gompertz model. In wild-type *H. alvei* H4, the growth ability is extremely strong. After the lag time of 1.01 h, it quickly enters the exponential phase with a maximum specific growth rate at 0.26. Through the knockout of the *luxI* and *luxR* genes, it is found that the growth ability was not obviously affected in the lag phase. However, the *luxI* and *luxR* genes had an influence on growth rate in the exponential phase and maximal biomass in the stationary phase. The V_max_ of mutants (around 0.21 h^−1^) was lower than wild and complementation types (0.26 h^−1^), especially the Δ*luxI* strain with a 23% descent rate. This result indicated that the QS system can only be triggered in the exponential phase when the microbial population density in the environment reaches the “quorum” threshold. *H. alvei* H4 also has a strong flagellar-dependent swimming ability and biofilm formation to ensure that it can move to seek a good nutritional matrix, excellently adhere to the food surface and resist antimicrobial substances. As shown in Figure 2B, *H. alvei* H4 flagella-dependent swimming is regulated by the *luxI* and *luxR* genes. The migration distance of the wild type increased rapidly with incubation time within 48 h and then remained constant by plate restriction, indicating that *H. alvei* H4 has a strong flagella-dependent swimming ability, while the strains without *luxI* or *luxR* genes were slightly inferior. Compared with the wild type, the migration distances of the Δ*luxI*, Δ*luxR* and Δ*luxIR* strains at 24 h were reduced by 50.0%, 64.3% and 54.3%, respectively. Furthermore, the biofilm formation ability of different phenotypes was evaluated by the crystal violet assay (Figure 2C). At 24 h, the biofilm yield of the *H. alvei* H4 mutant was significantly lower than that of the wild type. This phenomenon is similar to *Pseudomonas aeruginosa* [28] and *Acinetobacter baumannii* [29]. As the culture time prolonged, the differences between the wild type and the mutants in biofilm formation gradually increased. Thereby, the QS system is involved in the regulation of flagellar-dependent swimming ability and biofilm formation. These results also agree with Li’s work [30], where it is found that biofilm formation and swinging motility of *H. alvei* are regulated by the *lux*-type QS system.

### 3.3. Decarboxylase Detection

**Figure 3:**
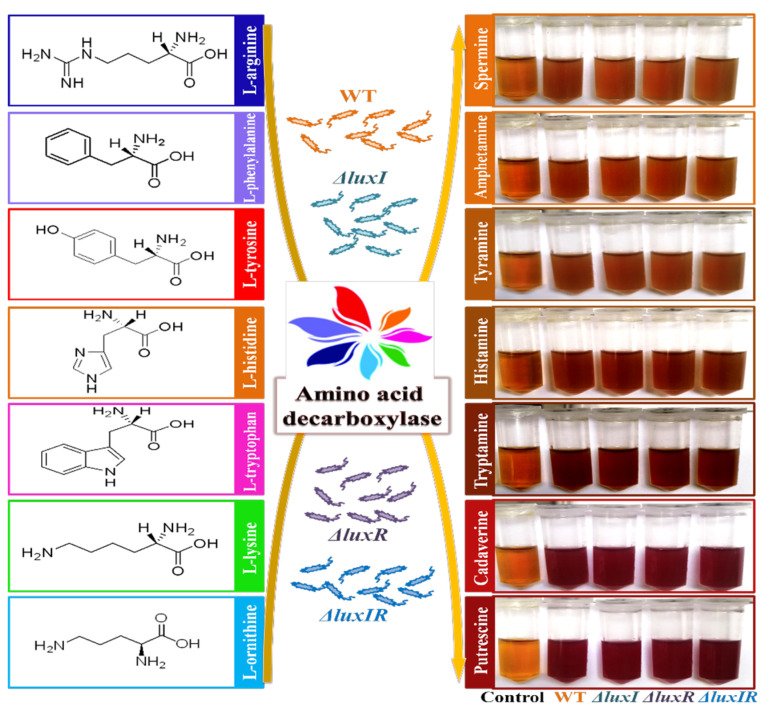
Decarboxylase detection of wild and mutant phenotypes by mediums with different precursor amino acids.

Wild and mutant phenotypes of *H. alvei* H4 strains were cultivated with a variety of precursor amino acids. A chromogenic reaction can distinguish whether the strain produces decarboxylase [31]. As shown in Figure 3, both mediums with L-ornithine and L-lysine precursors changed color from orange to deep red, while the color of other mediums did not change significantly, indicating *H. alvei* H4 has an ability to produce putrescine and cadaverine. Unfortunately, through this experiment, the difference in the yield of decarboxylase between the mutant strains and the wild strain cannot be discriminated by the decarboxylase chromogenic reaction. Therefore, HPLC experiments were carried out for further study.

### 3.4. Putrescine and Cadaverine Production

**Figure 4:**
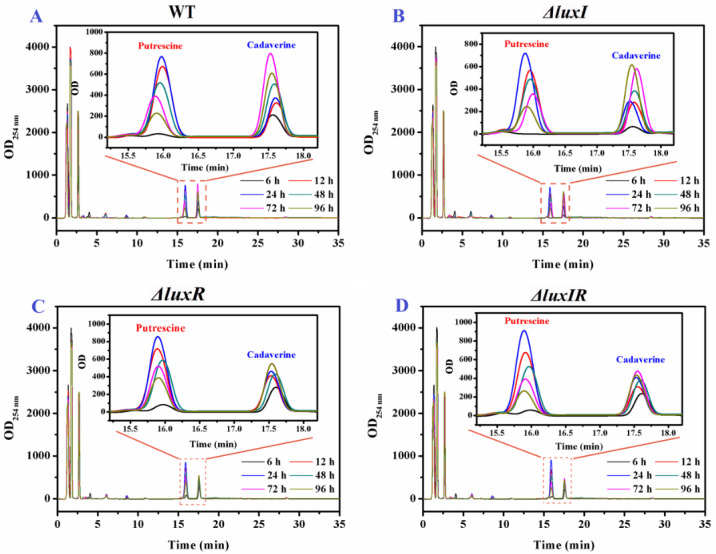
The HPLC results of the BAs from *H. alvei* H4 strains including wild and mutant phenotypes, (**A**) wild type, (**B**) Δ*luxI*, (**C**) Δ*luxR* and (**D**) Δ*luxIR*.

HPLC was applied to further quantitatively analyze the influence of *luxI* and *luxR* genes on putrescine and cadaverine production. As shown in Figure 4, two distinct high absorption peaks based on putrescine and cadaverine were found in the HPLC spectrum of biogenic amine production for each phenotypic strain of *H. alvei* H4. Through Figure 3 (Insert), it can be observed that the yield of the mutant strains of putrescine and cadaverine is significantly different from that of the wild type, especially cadaverine.

**Figure 5:**
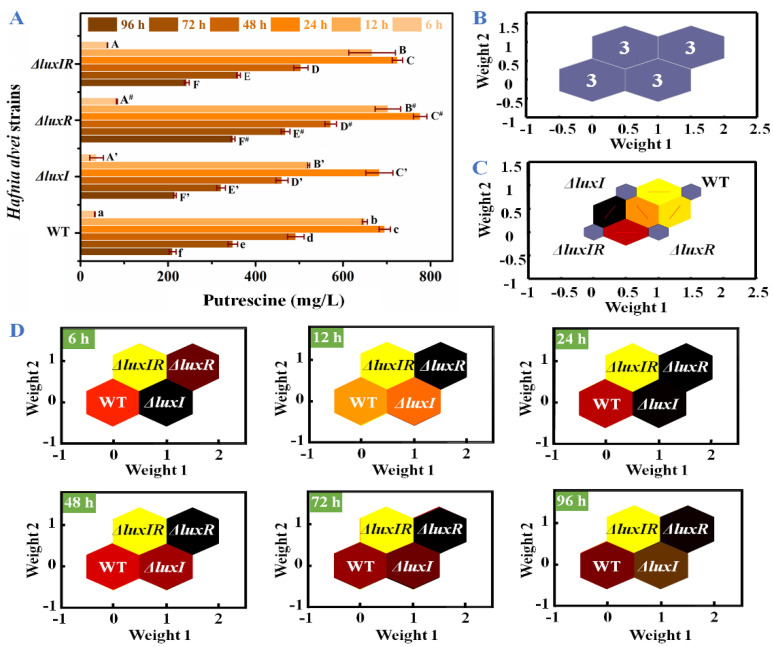
(**A**) The concentrations of putrescine, (**B**) visualized clustering results of SOM, (**C**) weight distances between adjacent neurons, (**D**) weight analysis of the wild-type and mutant phenotypes at different times.

The concentrations of putrescine were specifically analyzed in Figure 5A. All strains produced a large amount of putrescine in culture for 6–12 h. Compared with 6 h, the concentrations of putrescine in wild-type, Δ*luxI*, Δ*luxR* and Δ*luxIR* strains at 12 h increased by 19.7, 14.1, 8.44 and 10.8-fold, respectively. The maximum production and accumulation of putrescine in wild type, Δ*luxI*, Δ*luxR* and Δ*luxIR* were detected at 24 h, reaching up to 695.23 mg/L, 683.02 mg/L, 776.30 mg/L and 724.12 mg/L, respectively. For an in-depth analysis of the influence of *luxI* and *luxR* genes on putrescine productivity through data analysis, an identification model SOM including input layer and output layer was established. The input layer is a two-dimensional node matrix, where each node corresponds to a neuron representing the putrescine concentration of wild type, Δ*luxI*, Δ*luxR* and Δ*luxIR* throughout the culture (0–96 h). As shown in Figure 5B, the samples in the output layer were distinctly divided into four categories, and each category consisted of three samples belonging to wild type, Δ*luxI*, Δ*luxR* and Δ*luxIR*, respectively, without misclassified phenomena; hence, the accuracy rate of the prediction set was 100%. This demonstrated a significant difference in the production of putrescine between the various phenotypes. In Figure 5C, the topological function and distance function of the SOM are applied in 1000 iterations to describe the gap between the various phenotypic strains. The distance between wild type and Δ*luxI* and Δ*luxR* was bright yellow and dark yellow, respectively. The distance between Δ*luxIR* and Δ*luxR* is red, and the distance between Δ*luxIR* and Δ*luxI* is black, indicating that the Δ*luxR* phenotype is more distinct from the wild type in the putrescine accumulation process than the Δ*luxI* phenotype. In Figure 5D, the putrescine concentration profile is set to the modeled input, each neuron in the input layer is compared to each other neuron in the output layer by weight, and the dark to light color is applied depending on the magnitude of the weight [32]. Compared to the wild-type cluster, the Δ*luxI* cluster with the highest weight was black in the lag phase (6 h). After that, the weight of the Δ*luxR* cluster increased, replacing the Δ*luxI* cluster and turning into black from 12 h.

**Figure 6:**
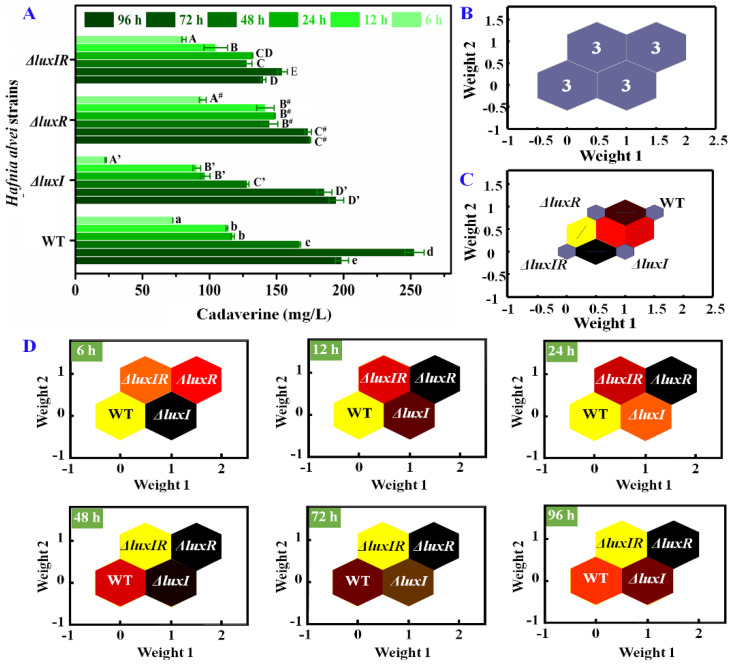
(**A**) The concentrations of cadaverine, (**B**) visualized clustering results of SOM, (**C**) weight distances between adjacent neurons, (**D**) weight analysis of the wild-type and mutant phenotypes at different times.

Likewise, the influence of the *luxI* and *luxR* genes on cadaverine production was specifically analyzed. As shown in Figure 6A, after 72 h of culture, the maximum concentration of cadaverine produced by wild strains was 252.7 mg/L, while the cadaverine concentrations of Δ*luxI*, Δ*luxR* and Δ*luxIR* were 194.5 mg/L, 175.1 mg/L and 154.2 mg/L, respectively, indicating that the *luxI* and *luxR* genes have a potential upregulation on the production of cadaverine. According to Wang’s work [26], similar results were obtained, where the amounts of putrescine and cadaverine of Δ*luxI* strains were always lower (*p* < 0.05) compared with wild-type *H. alvei* H4. The cadaverine concentrations of different phenotypic strains at 0–96 h were used as the basis neuron for SOM analysis in Figure 6B. According to the clustering property, it can be divided into four categories: wild type, Δ*luxI*, Δ*luxR* and Δ*luxIR*. The total identification accuracy is 100%. Through the self-organizing competition of the SOM network, the adjacent neurons were quantified. As shown in Figure 6C, the weight distances between neuron 1 of Δ*luxIR* and neuron 2 of Δ*luxI* was closest, suggesting that the cadaverine production process of the two is most similar. The weight distances between neuron 1 of Δ*luxIR* and neuron 4 of Δ*luxR* was most dissimilar. Moreover, the weight distance between wild type and Δ*luxI* was lighter than between wild type and Δ*luxR*, implying that *luxR* plays a more important role than *luxI* during the cadaverine production process. In further SOM analysis of different culture times as shown in Figure 6D, *luxI* plays a major role in the early stages of cadaverine production (6 h), and as the culture time prolongs (12–96 h), the role of the *luxR* gene gradually emerges.

In wild-type *H. alvei* H4, putrescine and cadaverine were mass-produced and accumulated during the exponential phase until the maximum was reached in the stationary phase. After that, putrescine and cadaverine were gradually consumed as available nitrogen sources, which may be due to a lack of nitrogen in the culture medium at the end of the culture period. Through comparative analysis of mutant and wild strains using SOM, it is not difficult to find that the knockout of any gene of *luxI* and *luxR* may significantly affect the production of putrescine and cadaverine, and the *luxR* gene has a greater impact. Similar to other corruption characteristics, the influence of the *luxI* gene on putrescine and cadaverine is mostly reflected in the lag phase, while the effect of the *luxR* gene is mainly concentrated in the exponential phase and stationary phase with a high density of bacteria. These results also agree with Yan’s work [33], where amino acid metabolism was associated with the *luxI/R* gene, which was also co-regulated in a growth phase-dependent manner. Choi et al. [34] also found that anthranilate metabolism was phase-dependently regulated by *las/rhl* quorum sensing in *Pseudomonas aeruginosa*. Anthranilate synthesis was especially activated by LasR in the log phase and repressed by RhlR; whereas, anthranilate degradation was repressed by LasR during the log phase and activated by RhlR in the late stationary phase. Bacterial quorum sensing (QS)-dependent gene expression is a dynamic response to cell density [35], thereby, it can be demonstrated in the effects of *lux*-type QS on spoilage characteristics in *H. alvei* H4. When *H. alvei* H4 grows in a lag phase with a slow growth rate and low cell density, the *luxI* gene is the main force of the QS system to regulate spoilage characteristics. According to Zhu’s work [36], the reason for this phenomenon may be that when the bacteria density is low, LuxI has to continuously strive to synthesize AHL signal molecules to achieve the threshold concentration and pair with the receptor protein LuxR to trigger the regulation of spoilage. With the growth of bacteria, the density of bacteria increases and the amount of AHL signal molecules is sufficient. The key point affecting QS regulation of spoilage characteristics, including mobility, biofilm formation and secretion of ornithine and lysine decarboxylase, is no longer the *luxI* gene but the *luxR* gene.

## 4. Conclusions

In this paper, the effects of the lux-type QS system on the growth characteristics and BA production of *H. alvei* H4 was explored. As the functions of the *luxI* and *luxR* genes are different, lux-type QS exhibited phase-differential regulation on growth, flagella-dependent swimming ability, biofilm formation and putrescine and cadaverine synthesis. In the lag phase, the population density is low, and *luxI* is the most critical factor affecting bacterial growth and BA production. Whereas, the *luxR* gene plays a major role in regulation of mobility, biofilm formation and production of putrescine and cadaverine in the exponential phase and stationary phase with a high density of bacteria. In short, the specific phase-dependent regulation mechanisms of the lux-type QS system on spoilage characteristics of *H. alvei* H4 and the transcriptional changes of *luxI* and *luxR* genes in the various growth phases (or different cell densities) and their functional characterizations will be subjects of our future research. The association of bacterial metabolism with the *luxI* and *luxR* genes still requires more research to determine the role of QS in *H. alvei*.

## Figures and Tables

**Table 1:** Bacterial strains and plasmids used in this study.

Bacterial Strain or Plasmid	Relevant Genotype or Description	Reference or Source
*H. alvei* H4	Genbank ID: GCA_008362885.1, wild-type strain	Isolated from spoiled instant sea cucumber by our lab [19]
Δ*luxI*	*H. alvei* H4 derivative, *luxI* mutant strain	This study
Δ*luxR*	*H. alvei* H4 derivative, *luxR* mutant strain	This study
Δ*luxIR*	*H. alvei* H4 derivative, *luxIR* mutant strain	This study
*comI*	*luxI* complementation strain, *luxI*- containing pUC19	This study
*comR*	*luxR* complementation strain, *luxR*-containing pUC19	This study
*comIR*	*luxIR* complementation strain, *luxIR*-containing pUC19	This study
*E.coli* TOP10	DH10b derivative of MG1655 (Genbank ID: GCA_000005845.2), recipient strain	Purchased from Takara
*E. coli* β2155	thrB1004 pro thi strA hsdS lacZ1M15 (F′ lacZ1M15 laclq traD36 proA+ proB+)1dap:: erm (Ermr))recA:: RPA-2-tet(Tcr)::Mu-km (Kmr) λpi [20] chloramphenicol resistance gene cassette (Cm^R^)	Purchased from Takara
PCVD442	Suicide plasmid, SacB, oriT, ampicillin resistance gene cassette (Am^R^)	Purchased from Songon
pUC 19	GenBank ID: M77789, Am^R^	Purchased from Takara
pET28a(+)/FaGH17A	GenBank ID: CDF79584.1, kanamycin resistance gene cassette (Km^R^)	Purchased from Takara

**Table 2:** Primers used for construction of mutant and complemented strains.

Sequence (5′-3′, Restriction Enzyme Sites are Underlined)	Restriction Enzyme
ATAGAATTCGTCGACATCACATTGATGTCAGACCTCAAGATTTC	EcoRI-SalI
ATAGGATCCATATCTGAGTGAGGATGAGCGAATTTATC	BamHI
TATGAATTCGTCGACATCAACATGCTCCCAATATCGCAC	EcoRI-SalI
TATGGATCCTTGGGCTCCTAGACGTTCAATTTCC	BamHI
ATAGGATCCATATGAATATCCTCCTTAGTTCCTATTC	BamHI
ATAGGATCCGAGCTGCTTCGAAGTTCCTA	BamHI

The underlined are restriction sites.

## Data Availability

The original contributions presented in the study are included in the article, further inquiries can be directed to the corresponding author.
